# The Prevention of Consumption

**Published:** 1893-03-04

**Authors:** 


### The Hospital

AN INSTITUTIONAL JOURNAL OF

Science, Medicine, Nursing, and Philanthropy.

*For Hospitals, Asylums, Infirmaries, Dispensaries, Medical Practitioners, Students, Nurses, and the Charitable Public.*

VOL. XIII.—No. 336.] [MARCH. 4, 1893.

### THE PREVENTION OF CONSUMPTION.

Is it really possible to prevent consumption? Let us answer this question by asking another: Is it really possible to prevent a wetting when it rains? We do not mean to say that everybody can avoid consumption by the use of proper means with as much certainty as everybody can avoid a wetting on a rainy day by the simple expedient of carrying a sufficiently large umbrella. There are no doubt persons so susceptible to consumption in one or other of its forms that they must die of it, do as they will. But these are the exceptions, the rare exceptions. For ordinary persons consumption is a communicable disease, which anybody may catch at any time, under conducive circumstances, but which the vast majority may as certainly avoid by the use of intelligent means.

Let us consider for a moment what consumption is according to modern scientific pathology. It is a destructive disease of the lungs or other parts of the body, set up by a small living organism known as the bacillus tuberculosis. That the bacillus tuberculosis is really the cause of consumption hardly admits of any doubt. The bacillus has so often been isolated outside the human body, cultivated outside the body generation after generation, used as an inoculating agent upon healthy animals so frequently and under all sorts of circumstances, with the practically invariable result of setting up tubercular phthisis of a deadly kind, that if anything at all of an experimental nature may be considered proved, the contention is demonstrated beyond question that the bacillus tuberculosis is the cause and origin of pulmonary and other forms of consumption. Besides all this we have living in our own and other civilised countries a body of men of such scientific competence and high personal character, that any conclusions which they declare to be demonstrations may for all practical purposes be considered final and irrefutable. And all these men declare with one voice that the bacillus tuberculosis is the cause of tubercular phthisis.

There are two main factors to be considered in the question of the prevention of consumption. These factors are, first, the bacillus tuberculosis, and, secondly, its victim; in other words, the arrow and its mark. Bring these together, and consumption may ensue; keep them apart, and consumption may be definitely prevented. These facts of science are of importance first of all to persons who are already consumptive. This conclusion has been intelligently recognized by the authorities of the North London Hospital for Consumption at Mount Vernon, Hampstead. The physicians there have issued a body of regulations for the guidance of their patients; and the first regulation reads thus: "Do not swallow your expectorations; this habit may lead to consumption of the bowels." That is to say, the expectorations of a consumptive person contain the bacillus tuberculosis, and if those expectorations be swallowed they will be passed by the stomach into the bowel, and will there find structures which provide them with a suitable nidus for their multiplication and destructive activity. But, it may be asked, if a person is already consumptive in the lungs, what is the use of preventing the infection of other organs? Is he not bound to die in any case? No, he is not! If an otherwise fairly healthy person, as is often the case, be merely suffering from one tuberculous centre located in one lung, he is very likely, under favourable circumstances, to recover. But if that same person, by the constant swallowing of his expectorations, charges his bowel with tubercle bacilli, and so sets up half a dozen other tuberculous centres in its twenty six feet of length, it is not only certain that he will die, but it is also equally certain that he will be the unconscious author of his own death. Here, then, is a kind of prevention which is to all intents and purposes a most important factor of cure. Consumptives everywhere should be warned against the serious risk and danger of swallowing their own expectorations.

But the prevention of consumption is, after all, much more an affair for the healthy than for those who are already consumptive; and especially is it a practical business of the most immediate urgency to those who are in daily contact with consumptives as nurses, as relatives, or as friends. On this point the regulations for patients and nurses at the Hampstead Consumption Hospital are of the most definite character. Thus they run: "Do not spit about the floor, nor into any utensil unless it contains a disinfectant. Do not spit about the streets. Indoors use a special spitting-cup or other vessel, in which is some disinfectant. Outdoors use a pocket-handkerchief or else use a piece of rag or paper, which must be burned as soon as you get home. If you spit into a pocket-handkerchief, it must be boiled for five minutes as soon as it is done with. Keep your rooms well aired, throw the window wide open when you leave the room, and always keep it open a little at the top all night. If there is a fireplace in the room, do not stop up the chimney. Keep your room clean; do not allow dust to remain on the floor. Consumptive patients should sleep alone. Mothers who are consumptive should not suckle their children."

All this, it is obvious, is directed to the one object of keeping the bacillus tuberculosis at a safe distance from healthy persons. It is considered proved, in short, that consumption is decidedly infectious, and that by a variety of means. We know, in the first place. that it is infectious by inoculation, because thousands of rabbits and guinea-pigs have been innoculated with tubercle bacilli, and in the result have died of phthisis. It is manifest, therefore, that if the expectoration of a tuberculous patient come into contact with a cut finger or an ulcerated or abraded surface of the skin or mucous membrane, the seeds of consumption may be sown in a moment, and death may finally ensue. But the best medical opinion at the North London Consumption Hospital and elsewhere considers, as we have already pointed out, that the swallowing also of the tubercule bacillus may result in phthisis. This is a second mode of infection; and there is also a third. The third is, perhaps, the most subtle and dangerous of all; and that is why the Mount Vernon authorities insist so strongly that all the expectoration of consumptive patients shall be disinfected or destroyed. This third mode of infection consists in the inhalation of tubercle bacilli, which are given off by the dried expectorations of consumptives. It is well known that if a consumptive patient spits about the floor, or about the streets, his expectorations, in a warm room or in the spring or summer air, rapidly dry, and so an apartment or a locality frequented by consumptives may be swarming with tubercle bacilli, which give no indication whatever of their presence either to sight, or taste, or smell. Hence the minuteness of the directions which tell attendants and friends to thoroughly boil pocket handkerchiefs which have been used by consumptives, and to burn rags or paper which may have been employed instead.

All these directions, it will be observed, are plain and practical. They are the natural outgrowth of scientific discovery and clinical observation and experience. Such rules, so arrived at, ought to be at once adopted universally both in hospitals and in private families. As a matter of fact, most hospitals have adopted them already. The difficulty is to get them carried into private houses, and, when there, to secure their intelligent application. The knowledge embodied in such rules is, at any rate, one of the fruits of modern scientific research, and its value to the world at large can never be overestimated.

